# Establishment of RPA-CRISPR/Cas12a Detection Methods for Rapid Largemouth Bass Ranavirus Surveillance

**DOI:** 10.3390/vetsci13080825

**Published:** 2026-08-18

**Authors:** Haoyu Wang, Yong Zhou, Peng Chen, Liping Zhang, Wen Zhu, Mingyang Xue, Yan Meng, Zhenyu Huang, Chen Xu, Yuding Fan, Chao Pei, Nan Jiang

**Affiliations:** 1 Division of Fish Disease, Yangtze River Fisheries Research Institute, Chinese Academy of Fishery Sciences, Wuhan 430223, China; 2 Engineering Lab of Henan Province for Aquatic Animal Disease Control, College of Fisheries, Henan Normal University, Xinxiang 453007, China; 3 Xinjiang Uygur Autonomous Region Fisheries Development Center (Xinjiang Uygur Autonomous Region Fisheries Research Institute), Urumqi 830000, China; 4 Chongqing Fisheries Technical Extension Center, Chongqing 401120, China

**Keywords:** largemouth bass, LMBRaV, RPA-CRISPR/Cas12a, RPA-CRISPR/Cas12a-LFD, rapid detection

## Abstract

The high mortality associated with largemouth bass ranavirus (LMBRaV) disease requires rapid and sensitive diagnosis methods to prevent virus spread and subsequent outbreak. The recombinase polymerase amplification combined with the CRISPR/Cas12a system (RPA-CRISPR/Cas12a) assay and RPA-CRISPR/Cas12a, coupled with lateral flow dipstick (RPA-CRISPR/Cas12a-LFD) assay for LMBRaV, were established in this study for low viral load surveillance. The major capsid protein (*mcp*) gene is highly conserved and is widely used as the target sequence in LMBRaV detection. First, based on the *mcp* gene sequence of LMBRaV, three candidate crRNAs were designed. Among them, the crRNA-2, which exhibited the highest cleavage activity, was selected through dual evaluation of fluorescence signal intensity and LFD color development. Then, the key reaction conditions for both assays were optimized as follows: LbCas12a protein concentration of 150 nM, crRNA-2 concentration of 200 nM, and ssDNA reporters’ concentration of 200 nM. Moreover, specificity evaluation showed that both combined assays specifically recognized LMBRaV, with no cross-reactivity detected against other aquatic pathogens, such as CyHV-2, GSIV, ISKNV, WSSV, GCRV II or CrERV. The detection limit was 1 copy/μL of DNA sample for both RPA-CRISPR/Cas12a and RPA-CRISPR/Cas12a-LFD assays. Finally, validation using 24 clinical samples (16 positive, eight negative) showed that both LMBRaV RPA-CRISPR/Cas12a assays achieved 100% detection rate, whereas conventional PCR detected 13 positive samples (detection rate 81.26%). ddPCR served as the reference method for clinical validation. Therefore, the LMBRaV RPA-CRISPR/Cas12a assay and the RPA-CRISPR/Cas12a-LFD assay provide sensitive, specific, and easy-to-operate methods for the rapid detection of LMBRaV.

## Simple Summary

In this study, the RPA-CRISPR/Cas12a assay and the RPA-CRISPR/Cas12a-LFD assay were established for largemouth bass ranavirus (LMBRaV) detection. Optimization of the crRNA and reaction conditions led to the selection of crRNA-2, with optimal concentrations established as 150 nM for LbCas12a, 200 nM for crRNA-2, and 200 nM for ssDNA reporters. Both assays exhibited excellent specificity, detecting only LMBRaV, and achieved a sensitivity of 1 copy/μL of DNA sample. Moreover, both assays achieved higher detection rates than conventional PCR assays. These two assays provide convenient and reliable methods for LMBRaV surveillance.

## 1. Introduction

Largemouth bass (*Micropterus salmoides*) is native to North America [1] and has been widely farmed in many provinces in China now [2]. However, viral diseases, which cause high mortality, have threatened largemouth bass aquaculture and are difficult to prevent and treat. Among them, largemouth bass ranavirus (LMBRaV) disease (LMBVD) poses the most serious challenge [3,4].

LMBRaV, belonging to the genus *Ranavirus* within the family Iridoviridae, is a double-stranded DNA virus with icosahedral capsids [5]. Since the first isolation in Lake Weir, Florida, USA in 1991 [6], LMBRaV has spread to 22 states in the USA and then to Asia [7]. Since 2006, the virus has frequently broken out in Guangdong, Zhejiang, Hubei and other regions of China, with mortality rates reaching as high as 100% and causing severe economic losses [8,9,10].

As we all know, disease prevention relies mainly on prophylaxis and interruption of transmission routes [11,12]. The clinical symptoms of LMBRaV infection varied depending on the route of infection, fish age, viral strain virulence and water temperature; moreover, different passage generations also exhibited significant variations in pathogenicity and tissue tropism [5,13,14,15,16,17,18]. In the early stage of infection, some diseased fish exhibited behavioral abnormalities (such as floating at the water surface and lethargy) and hemorrhagic spots or ulcers on the body surface, while some diseased fish did not show any symptom which led to disease transition [1,10,19]. Therefore, the establishment of sensitive, specific, simple and rapid detection methods is crucial for the early diagnosis of LMBRaV.

At present, the detection of LMBRaV mainly relied on nucleic acid amplification methods, including conventional PCR [7,20], loop-mediated isothermal amplification (LAMP) and quantitative real-time PCR [21,22]. As a conserved gene, the major capsid protein (*mcp*) gene normally serves as the amplification target gene [3,12,23,24]. However, the sensitivity of these methods was limited for detection of samples at low viral loads. Jiang et al. established a droplet digital PCR (ddPCR) method for LMBRaV, achieving a detection limit of 2 copies/μL [24]. However, this method was not suitable for rapid detection due to its instrumentation demands and long processing time.

In 2006, the recombinase polymerase amplification (RPA), an isothermal nucleic acid amplification technique, was established [25]. It could rapidly amplify DNA at a constant temperature (37–42 °C) and has been widely used in the detection of bacteria, viruses, parasites and food safety [26]. RPA offered the advantages of simple operation, less requirements than thermal cycling instruments, and high amplification efficiency. Additionally, it combined with lateral flow dipsticks (LFDs) and realized visual diagnosis [3]. In 2022, Zhu et al. established an RPA rapid detection technique for LMBRaV with a detection limit of 89 copies/μL and a detection time shortened to within 30 min [26]. In 2026, Wang et al. selected optimized primers and probes, and developed more sensitive visualization RPA assays for LMBRaV detection with the detection limit of 1 copies/μL [3]. However, RPA used alone might suffer from false positives due to non-specific amplification. The CRISPR/Cas12a system (also known as Cpf1) belonged to the class 2 type V CRISPR-Cas system and required only a single crRNA for target recognition [27]. Upon activation by target DNA, the Cas12a protein not only exhibited cis-cleavage activity to cut the target double-stranded DNA, but also initiated non-specific trans-cleavage activity, indiscriminately cutting any single-stranded DNA molecules present in the system [28]. Based on this characteristic, the combination of RPA with the CRISPR/Cas12a system enabled an integrated “amplification-recognition-signal amplification” design: RPA rapidly enriches trace amounts of target DNA and the Cas12a-crRNA complex specifically recognizes the amplicon and activates trans-cleavage, which then cleaves fluorescent reporters or LFD reporters to generate detectable signals. This combined strategy not only overcame the problems of insufficient sensitivity and false positives associated with RPA alone, but also retained the simplicity of isothermal amplification, and showed excellent performance in aquatic pathogen detection in recent years [29,30,31,32,33]. To date, the RPA-CRISPR/Cas12a system has been applied for rapid and highly sensitive detection of several aquatic pathogens, such as Chinese rice-field eel rhabdovirus, Tilapia parvovirus, *Pseudomonas plecoglossicida* and irodovirus in large yellow croaker, and ranavirus and infectious spleen and kidney necrosis virus in mandarin fish [34,35,36,37,38]. Although an RPA-CRISPR/Cas12a detection method for LMBRaV has been reported with a sensitivity of 50 copies/μL [39], the detection limit was not low enough, which might lead to missed diagnoses and disease transition. Hence, more sensitivity methods are still needed. Because of the high sensitivity, the CRISPR/Cas12a system could examine largemouth bass with ultralow viral load—including asymptomatic carriers—within a limited detection time.

Based on our previously established RPA and RPA-LFD methods for LMBRaV [3], this study combined RPA assay with the CRISPR/Cas12a system to reduce the false positives and improve detection sensitivity. After screening of crRNA and optimization of the reaction system, the sensitivity, specificity and clinical samples detection rate of LMBRaV RPA-CRISPR/Cas12a assay and RPA-CRISPR/Cas12a-LFD visual assay were both analyzed. The aim was to provide a specific and easy-to-operate technique for the rapid diagnosis of low-virus-load LMBRaV.

## 2. Materials and Methods

### 2.1. Virus and Viral DNA Preparation

Largemouth bass ranavirus LMBRaV-HB001 was isolated from diseased largemouth bass in Hubei Province and stored in the Yangtze River Institute of Fisheries Research of the Chinese Academy of Fisheries Sciences [10]. The virus was confirmed by phylogenetic analysis and electron microscopy observation. Other aquatic viruses, Cyprinid herpesvirus II (CyHV-2), Chinese giant salamander iridovirus (GSIV), infectious spleen and kidney necrosis virus (ISKNV), white spot syndrome virus (WSSV), grass carp reovirus II (GCRV II) and Chinese rice eel rhabdovirus (CrERV), were obtained from the Yangtze River Institute of Fisheries Research of the Chinese Academy of Fisheries Sciences [3,24,29,40,41].

Viral DNA of LMBRaV, CyHV-2, GSIV, WSSV and ISKNV was prepared from infected cells/tissues using the Viral DNA Extraction Kit (Omega Bio-tek, Norcross, GA, USA) according to the manufacturer’s instructions; the extracted DNA was eluted in 50 μL of elution buffer. Viral RNA of GCRV II and CrERV was processed using the Total Cell/Tissue RNA Extraction Kit (YEASEN, Shanghai, China) and then reverse-transcribed into cDNA samples according to the manufacturer’s instructions. cDNA samples were used in the Cas12a-based assay. All the DNA/cDNA samples for specificity were tested by ddPCR or qPCR, and then stored at −20 °C.

In this study we used two negative controls. The negative control in the kit was used to demonstrate that there was no contamination of the reagents, etc. The healthy largemouth bass genome was extracted by Tissue DNA Extraction Kit (Omega Bio-tek, Norcross, GA, USA) and used to demonstrate that the reaction was unable to amplify a product from the host genome.

### 2.2. Design of crRNA and ssDNA Reporter for RPA-CRISPR/Cas12a

Based on our previous study [3], the optimal LMBRaV basic-RPA primers (F6/R6, Table 1) targeting the *mcp* gene was selected and used for subsequent experiments. The crRNA1/2/3 were designed based on the amplification region of the F6/R6 (Figure S1). The crRNAs consisted of a universal scaffold sequence at the 5′ end and a target-specific spacer sequence at the 3′ end. The standard scaffold sequence of Cas12a crRNA was 5′-UAAUUUCUACUAAGUGUAGAU-3′ (Table 1), which was responsible for binding to the Cas12a protein to form a ribonucleoprotein complex. The crRNAs were designed using SnapGene software. The three crRNAs had spacer lengths ranging from 21 to 23 nt, with GC contents of 52.4%, 59.1%, and 65.2%, respectively. All crRNAs were synthesized by Aoke (Wuhan) Biotechnology Company Limited(Wuhan, China).

To visualize the trans-cleavage activity of Cas12a, two types of ssDNA reporters were designed and synthesized: an LFD-ssDNA reporter (5′-6FAM-TTATT-Biotin-3′) for lateral flow dipstick detection, and an ssDNA reporter (5′-6FAM-TTATT-BHQ1-3′) for fluorescence detection (Table 1). When intact, the reporter’s fluorescence signal was quenched or it was captured by the control line; upon target activation, Cas12a cleaved the reporter, generating a detectable fluorescence signal or allowing capture by the test line. All ssDNA reporters were synthesized by Aoke (Wuhan) Biotechnology Company Limited.

### 2.3. Reaction Systems of RPA-CRISPR/Cas12a Assay and RPA-CRISPR/Cas12a-LFD Visual Assay

The basic-RPA reaction (50 μL) was prepared following the instructions of the basic-RPA kit (GENENODE, Wuhan, China) using: 32.9 μL A buffer, 2 μL forward and reverse primers (10 μM), 8.6 μL ddH_2_O, 2 μL nucleic acid templates, and 2.5 μL B buffer. The mixture was incubated at 38 °C for 25 min [3]. Then, 1 μL of the basic-RPA product was used as the target DNA and added to the CRISPR/Cas12a trans-cleavage reaction system. The reaction was carried out using the optimized HOLMES buffer system (TOLOBIO, Shanghai, China) in a total volume of 20 μL, with the specific components shown in Table 2. The components listed in Table 2 were mixed thoroughly and placed in a real-time fluorescence PCR instrument. The reaction was incubated at 37 °C for 30 min according to the LbCas12a (CP1) Nuclease product manual in a QuantStudio Real-time PCR (Yeasen, China), and fluorescence values in the FAM channel were collected every 30 s. Post-incubation, endpoint fluorescence was quantified and results were visually confirmed using a blue-light transilluminator. Healthy largemouth bass genomic DNA or the kit negative control served as the negative control, and nuclease-free water was used as the blank control. A sample was considered positive when its fluorescence value was significantly higher than that of the negative control. The reactions were performed in duplicate for each sample.

To achieve on-site rapid visual detection, an RPA-CRISPR/Cas12a-LFD detection system was established by combining RPA-CRISPR/Cas12a with LFD. A volume of 1 μL of the basic-RPA product described above was added to the CRISPR/Cas12a trans-cleavage reaction, and the reaction composition was essentially the same as that of the fluorescence assay, except that the ssDNA reporter was replaced with a dual-labeled LFD-ssDNA reporter (5′-6FAM-TTATT-Biotin-3′). The detailed components are shown in Table 3. The mixture was mixed and incubated in a constant-temperature metal bath at 37 °C for 20–30 min to ensure sufficient cleavage of the reporter by Cas12a. Then, 10 μL of the reaction product was mixed with 40 μL of nuclease-free water, and then the dipstick (TOLOBIO, Shanghai, China) was inserted into the mixture at room temperature for 5–10 min until the control line clearly appeared. The results were considered positive when both the control line and the test line appeared; negative when only the control line appeared; and invalid when no lines appeared or only the test line appeared. For the LFD assay, the results were directly determined by visual inspection. The reactions were performed in duplicate for each sample.

### 2.4. Screening of crRNA for RPA-CRISPR/Cas12a and RPA-CRISPR/Cas12a-LFD Assays

The crRNA1/2/3 activities were screened and optimized using both the fluorescence detection system and LFD detection system. The fluorescence values were quantified by a real-time PCR and results were visually confirmed using a blue-light transilluminator, and the LFD results were directly determined by visual inspection. Fluorescence values and test-line color intensity on the dipstick were used as the evaluation index. Three independent replicate experiments were performed.

### 2.5. Optimization of the Concentration of LbCas12a Protein, crRNA and ssDNA Reporters

To achieve optimal detection conditions, the optimization focused on the concentration of LbCas12a protein, crRNA, ssDNA fluorescence reporter, and LFD-ssDNA reporter. The reaction temperature and time have been optimized previously [3]. The fluorescence values were quantified by a real-time PCR and results were visually confirmed using a blue-light transilluminator, and the LFD results were directly determined by visual inspection. Fluorescence values (for the fluorescence detection system) or test-line color intensity on the dipstick (for the LFD system) were used as the evaluation index. The concentration ranges (25–250 nM) for LbCas12a protein, crRNA, and ssDNA reporters were set according to the manufacturer’s instructions (TOLOBIO, Shanghai, China).

Gradient concentrations of LbCas12a protein (25, 50, 100, 150, 200, 250 nM) were set under the following fixed conditions: 250 nM crRNA-2, 250 nM ssDNA fluorescence reporter, 1 μL basic-RPA product, and the total reaction volume was 20 μL. Under the optimal LbCas12a concentration, gradient concentrations of crRNA-2 (25, 50, 100, 150, 200, 250 nM) were tested using 250 nM ssDNA fluorescence reporter and 1 μL basic-RPA amplification product. Finally, gradient concentrations of the ssDNA fluorescence reporter (25, 50, 100, 150, 200, 250 nM) were tested using the optimal LbCas12a and crRNA-2 concentration, with the basic-RPA product as the target. All reactions were incubated at 37 °C for 30 min and the fluorescence values were monitored. Three independent replicate experiments were performed.

To achieve visual detection using dipsticks, a dual-labeled LFD-ssDNA reporter (5′-6FAM-TTATT-Biotin-3′) was employed. The signal output mode of the LFD assay differed from that of the fluorescence assay. The reporter concentration should ensure a clear test line color after cleavage while avoiding background colors caused by intact reporters. Under the optimal LbCas12a and crRNA-2 concentration, gradient concentrations of the LFD reporter (25, 50, 100, 150, 200, 250 nM) were set. Using basic-RPA product as the target, the reaction was incubated at 37 °C for 30 min, and then 10 μL of the product was collected for LFD detection. Three independent replicate experiments were performed.

### 2.6. Specificity and Sensitivity Evaluation of RPA-CRISPR/Cas12a and RPA-CRISPR/Cas12a-LFD Assays

The specificity and sensitivity of the RPA-CRISPR/Cas12a and RPA-CRISPR/Cas12a-LFD assays was validated under the optimized conditions. The DNA/cDNA samples of CyHV-2, GSIV, WSSV, ISKNV, GCRV II, and CrERV were used to evaluate the specificity. Among them, ISKNV was the other major viral pathogen affecting largemouth bass.

LMBRaV viral DNA samples were absolutely quantified by droplet digital PCR [24] to obtain viral DNA copy numbers. A volume of 2 μL of standard DNA sample was mixed with 10 μL of dPCR EvaGreen SurperMix (Bio-Rad), 0.2 μL each of forward and reverse primers, and 7.6 μL of nuclease-free water (total volume was 20 μL). The droplets were generated by the Bio-Rad QX200™ let Generator (Bio-Rad, Hercules, CA, USA). After PCR amplification, the droplets were analyzed by the Bio-Rad QX200™ Droplet Reader (Bio-Rad, Hercules, CA, USA), and the virus copy number was calculated based on the number of positive droplets. The standards were serially diluted 10-fold to generate a series of templates at concentrations of 10^5^, 10^4^, 10^3^, 10^2^, 10, 1, and 0.5 copies/μL. Healthy largemouth bass genome DNA was used as the control. Three replicates were set for each concentration, and three independent replicate experiments were performed.

### 2.7. Practical Application Evaluation of RPA-CRISPR/Cas12a and RPA-CRISPR/Cas12a-LFD Assays

To evaluate the detection performance of the RPA-CRISPR/Cas12a and RPA-CRISPR/Cas12a-LFD assays in clinical samples, 24 largemouth bass samples (including asymptomatic and acute infection stage) from multiple farms were selected (including 14 samples used in Wang et al. 2026) [3]. A total of 100 ± 10 mg tissue was collected for virus DNA extraction. Initially, all the samples were quantitatively determined by ddPCR and subsequently subjected to parallel detection using the RPA-CRISPR/Cas12a assay, the RPA-CRISPR/Cas12a-LFD visual assay, and the conventional PCR assay [42].

### 2.8. Statistical Analysis

The data were expressed as mean ± standard deviation (SD) values from three independent replicates and were analyzed using GraphPad Prism 6.0 (one-way ANOVA and Dunnett’s test). Normality and homogeneity of variance were tested before ANOVA. The significance levels were denoted as * *p* < 0.05 and ** *p* < 0.01.

## 3. Results

### 3.1. Screening of crRNAs for RPA-CRISPR/Cas12a and RPA-CRISPR/Cas12a-LFD Assays

**Figure 1:**
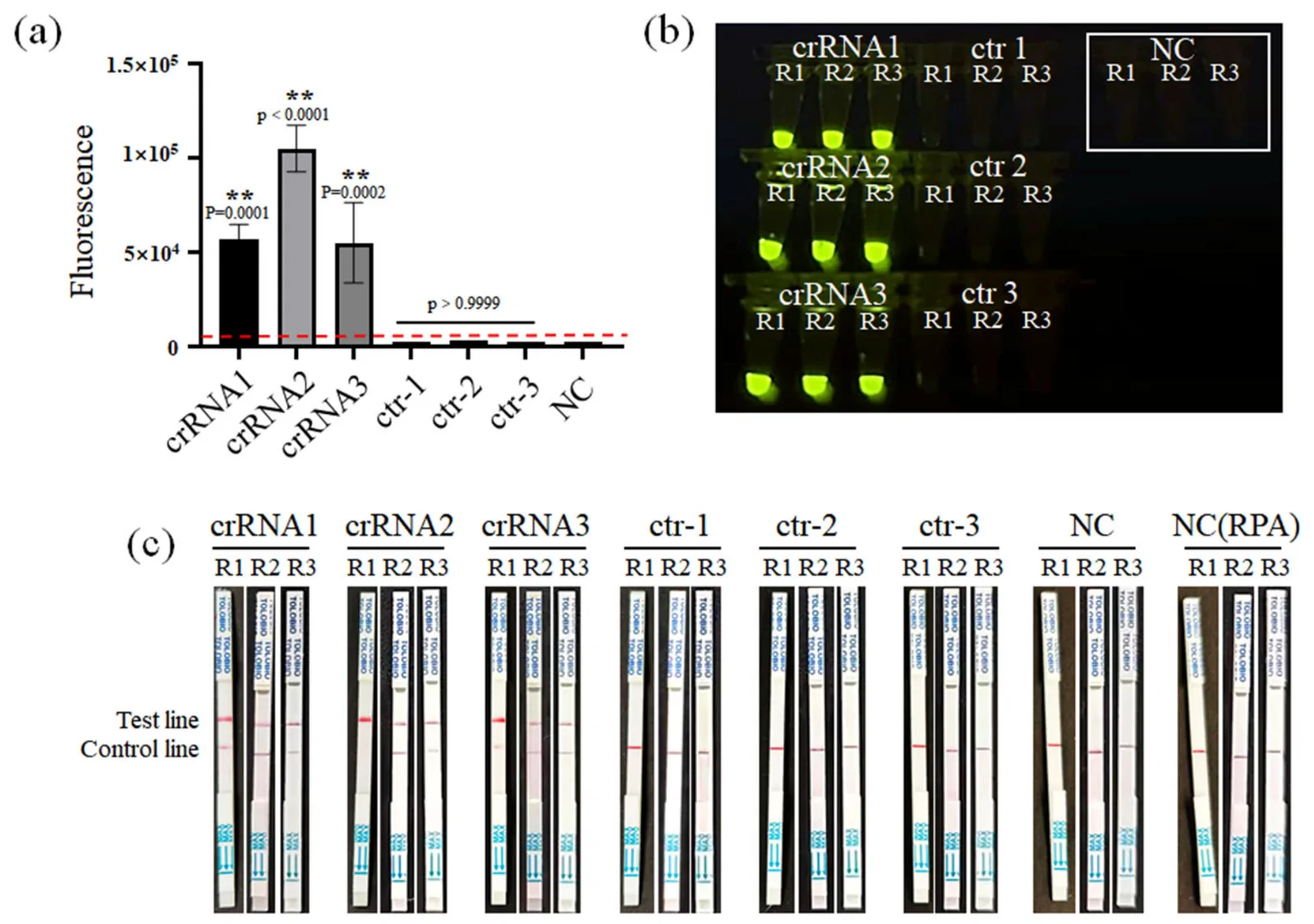
CrRNAs screening. (**a**) Fluorescence intensity histogram of crRNA1-3; (**b**) fluorescence-based screening of crRNA1-3; (**c**) lateral flow dipstick-based screening of crRNA1-3. NC (RPA): negative control provided by the kit, NC: nuclease-free water. Three independent replicate experiments were performed. Data represent mean ± SD (*n* = 3), ** *p* < 0.01. The red dashed line shows the cut-off value (negative control mean ± SD).

Three crRNAs were designed and screened. The fluorescence signal intensity of the three crRNAs showed that crRNA-2 exhibited the highest endpoint fluorescence intensity (Figure 1a,b) (*p* < 0.0001). No significant increased fluorescence signal was detected in the negative control (NC) groups. A fluorescence signal intensity higher than the cut-off value was considered as positive. The cut-off value was defined as the mean ± SD of the negative controls. The results of three independent replicate experiments were consistent, with the fluorescence values ranking as follows: crRNA-2 (56,982.3 ± 8090.9) > crRNA-1 (105,227 ± 12,330) > crRNA-3 (41,631.7 ± 38,444.7). No significant increased fluorescence signal was detected in the control crRNA1-3 groups (without crRNA).

Moreover, the CRISPR/Cas12a cleavage products from the same template were subjected to LFD detection, and the color intensity of the test line indicated that crRNA-2 was the clearest, although three crRNAs showed relatively similar color development (Figure 1c). Only the control line presented in the NC and control crRNA1-3 groups. The color intensity was consistent across three independent replicate experiments.

Therefore, crRNA-2, which exhibited the highest cleavage activity and the best detection signal, was selected as the optimal crRNA for subsequent experiments.

### 3.2. Optimization of Concentration of LbCas12a Protein, crRNA and ssDNA Reporters

**Figure 2:**
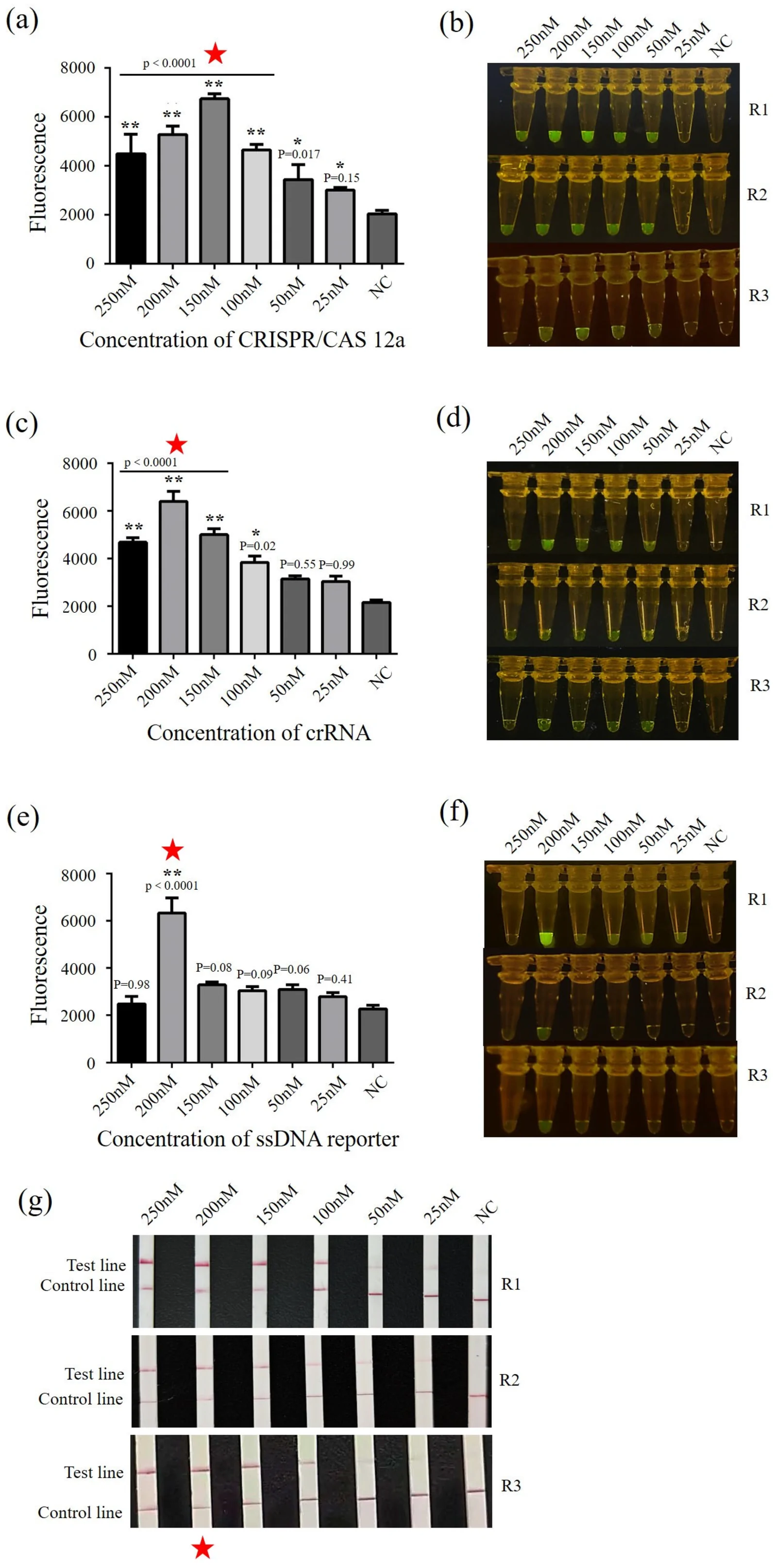
Optimization of detection conditions. (**a**) Fluorescence intensity histogram for optimal LbCas12a concentration (25, 50, 100, 150, 200, 250 nM) screening; (**b**) fluorescence image for optimal LbCas12a concentration screening; (**c**) fluorescence intensity histogram for optimal crRNA concentration (25, 50, 100, 150, 200, 250 nM) screening; (**d**) fluorescence image for optimal crRNA concentration screening; (**e**) fluorescence intensity histogram for optimal ssDNA reporter concentration (25, 50, 100, 150, 200, 250 nM) screening; (**f**) fluorescence image for optimal ssDNA reporter concentration screening; (**g**) optimal concentration screening of LFD-ssDNA reporter (25, 50, 100, 150, 200, 250 nM) using lateral flow dipstick assay. Star indicates the selected optimal concentrations. NC: nuclease-free water. Three independent replicate experiments were performed. Data represent mean ± SD (*n* = 3), * *p* < 0.05, ** *p* < 0.01.

LbCas12a protein was diluted to 25–250 nM, and the fluorescence signal intensity was analyzed. The signal intensity was gradually increased with the increasing LbCas12a concentration. When the concentration reached 150 nM, the fluorescence signal reached the peak (*p* < 0.0001), then decreased from 150 to 250 nM (Figure 2a,b).

crRNA-2 was diluted to 25–250 nM, and the reaction was performed under LbCas12a concentration of 150 nM. The fluorescence signal intensity results showed that the signal intensity reached the highest level at a crRNA concentration of 200 nM (*p* < 0.0001), followed by a decline at higher concentrations (Figure 2c,d).

Under the optimized conditions of LbCas12a (150 nM) and crRNA-2 (200 nM), the concentration the ssDNA fluorescence reporter (5′-6FAM-TTATT-BHQ1-3′) and LFD-ssDNA reporter were both set at was 25–250 nM. Post-reaction, the results showed that the fluorescence signal intensity increased and reached its maximum at a concentration of 200 nM (*p* < 0.0001) and decreased at higher concentration. For the LFD assay, the color intensity of the test line was increased from 25 nM to 250 nM, and the sample at the concentration of 200 nM displayed the highest color intensity on the test line and lower color intensity on the control line. Therefore, the optimal working concentration of the ssDNA fluorescence reporter and LFD-ssDNA reporter were both determined to be 200 nM (Figure 2e–g).

Hence, the optical concentrations of LbCas12a protein (150 nM), crRNA (200 nM), and ssDNA reporters (200 nM) were used for subsequent experiments.

### 3.3. Specificity of RPA-CRISPR/Cas12a and RPA-CRISPR/Cas12a-LFD Assays

**Figure 3:**
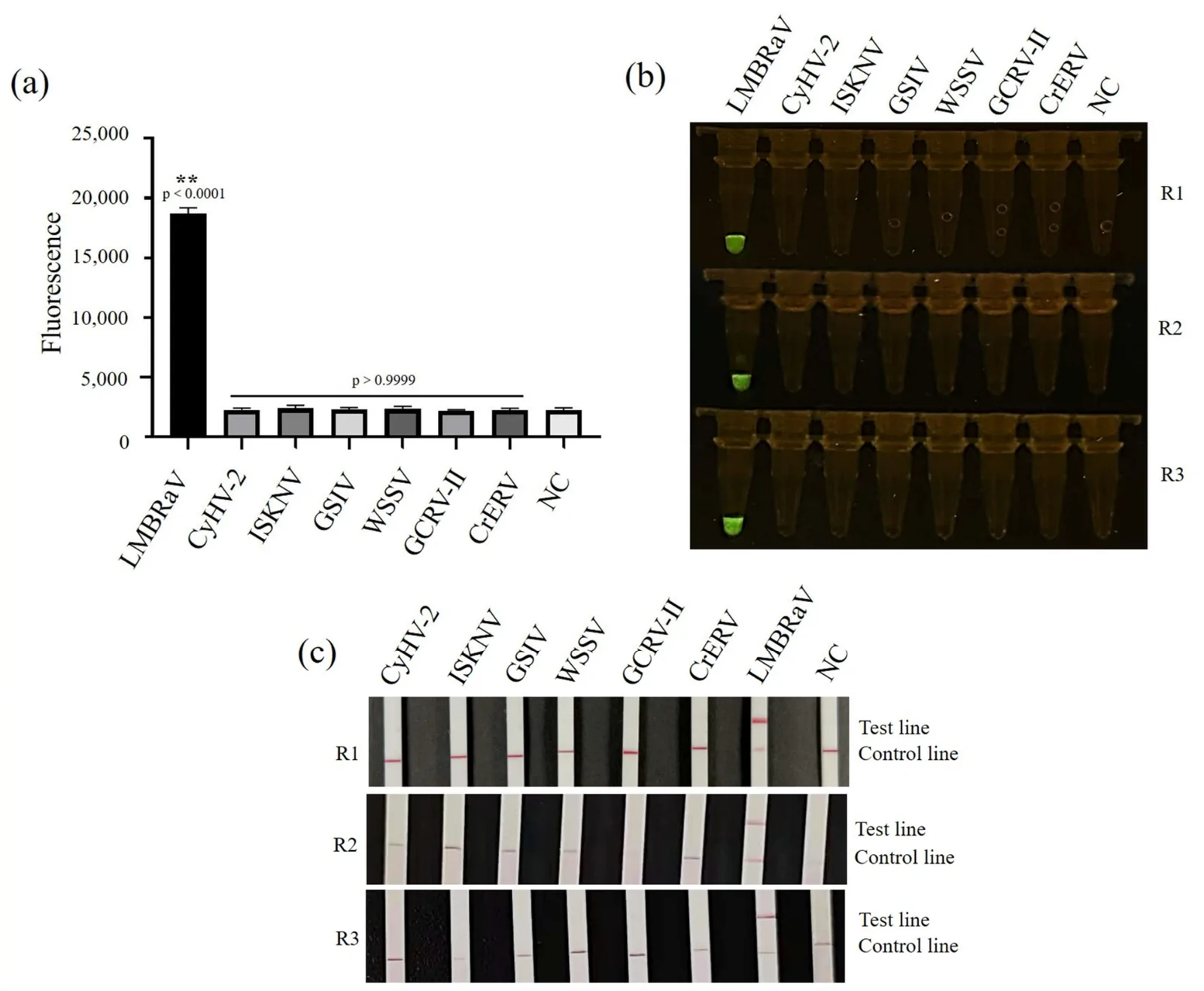
Specificity of RPA-CRISPR/Cas12a and RPA-CRISPR/Cas12a-LFD assays. Virus DNA/cDNA of LMBRaV, CyHV-2, ISKNV, GSIV, WSSV, GCRV-II, and CrERV were tested. (**a**) Fluorescence intensity histogram for specificity evaluation; (**b**) fluorescence image for specificity evaluation; (**c**) lateral flow dipstick-based specificity evaluation. NC: genomic DNA from healthy fish. Three independent replicate experiments were performed. Data represent mean ± SD (*n* = 3), ** *p* < 0.01.

The nucleic acid samples of LMBRaV, CyHV-2, GSIV, ISKNV, GCRV II, CrERV, WSSV were used to identify the specificity of the fluorescence and LFD detection system. Only the LMBRaV virus DNA sample exhibited a significant increased fluorescence signal post RPA-CRISPR/Cas12a reaction (*p* < 0.0001), as well as the endpoint fluorescence value, which was significantly higher than that of the negative control (Figure 3a,b). In contrast, no increased fluorescence signal was observed for CyHV-2, GSIV, ISKNV, GCRV II, CrERV, WSSV, or healthy largemouth bass.

After the reactions of the LFD assay, only the dipstick for the LMBRaV positive sample displayed remarkable test line and control line. For nucleic acid samples of CyHV-2, GSIV, ISKNV, GCRV II, CrERV, WSSV and healthy largemouth bass genomic DNA, only control line was developed, which indicated the reactions were successfully run but no positive signal was presented (Figure 3c).

The results indicated that the LMBRaV RPA-CRISPR/Cas12a assay and LFD detection assay were both highly specific for LMBRaV, with no cross-reactivity against other viruses or the host genome.

### 3.4. Sensitivity of RPA-CRISPR/Cas12a and RPA-CRISPR/Cas12a-LFD Assays

**Figure 4:**
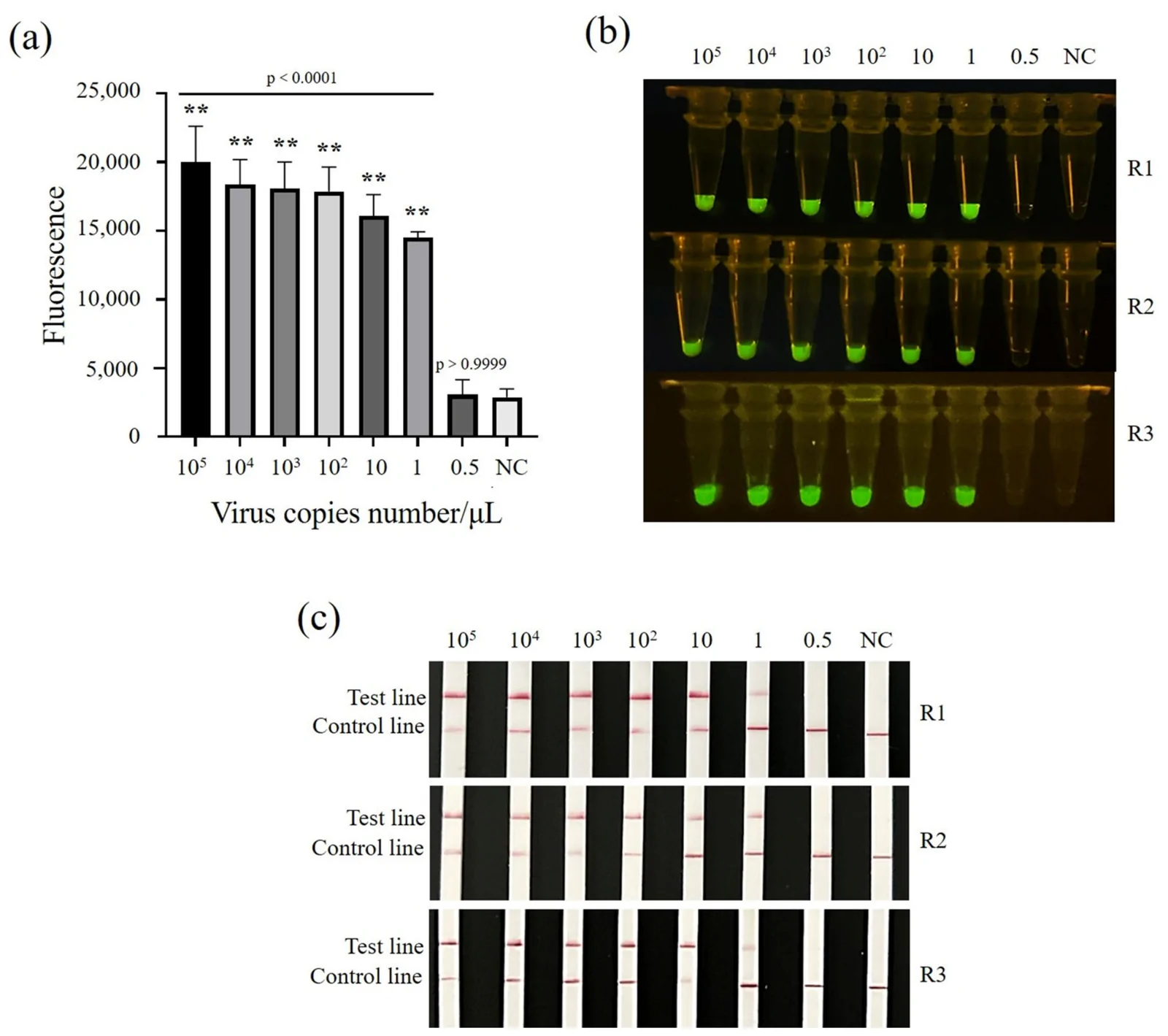
Sensitivity evaluation of LMBRaV RPA-CRISPR/Cas12a and RPA-CRISPR/Cas12a-LFD assays. Ten-fold dilutions of LMBRaV virus DNA (10^5^, 10^4^, 10^3^, 10^2^, 10, 1, and 0.5 copies/μL) were tested. (**a**) Fluorescence intensity histogram for sensitivity evaluation; (**b**) fluorescence image for sensitivity evaluation; (**c**) lateral flow dipstick-based sensitivity evaluation. NC: nuclease-free water. Three independent replicate experiments were performed. Data represent mean ± SD (*n* = 3), ** *p* < 0.01.

Serially diluted LMBRaV viral DNA templates at concentrations of 10^5^, 10^4^, 10^3^, 10^2^, 10, 1, and 0.5 copies/μL were used to determine the sensitivity of two assays. Healthy largemouth bass DNA served as the negative control. As shown in Figure 4a,b, when the template concentration ranged from 10^5^ to 1 copy/μL, the fluorescence signals were significantly higher than the negative control (*p* < 0.0001), while no appreciable signal above the negative control level was detected for the 0.5 copy/μL samples (Figure 4a,b). A concentration of 1 copy/μL was consistently detected across three independent replicates. Therefore, the detection limit of the RPA-CRISPR/Cas12a assay was determined to be 1 copy/μL.

To validate the sensitivity of Cas12a-LFD assays, the above diluted templates were tested under optimal conditions. The test line was observed in samples with concentrations ranging from 10^5^ to 1 copy/μL, and the color intensity gradually decreased with decreasing template concentration. No test line was detected for the sample at a concentration of 0.5 copy/μL, as well as the negative control (Figure 4c). A concentration of 1 copy/μL was consistently detected across three independent replicates (Figure 4c). Therefore, the visual detection limit of the RPA-CRISPR/Cas12a-LFD assay was determined to be 1 copy/μL.

### 3.5. Practical Application Evaluation of RPA-CRISPR/Cas12a and RPA-CRISPR/Cas12a-LFD Assays

Twenty-four samples were selected for practical application evaluation. The virus loads were detected by ddPCR [24], and eight samples and blank controls tested negative for the virus and 16 samples tested positive (Table 4 and Table 5). The conventional LMBRaV PCR method [42] detected 13 positive samples, but three low-copy samples with viral copy numbers below 760 copies/μL were not detected (Table 4, Figure S2). The detection rate was 81.26% compared to the ddPCR results. RPA-CRISPR/Cas12a and RPA-CRISPR/Cas12a-LFD assays consistently detected 16 positive samples, which agree with the ddPCR results. Therefore, the detection rate of RPA-CRISPR/Cas12a and RPA-CRISPR/Cas12a-LFD assays were 100% compared to the ddPCR results.

## 4. Discussion

LMBRaV causes large-scale outbreaks of disease and mass mortality in largemouth bass, and infected fish can become virus carriers, making disease prevention and control difficult [5,12,22]. Therefore, early surveillance of LMBRaV is particularly important and urgent [26]. Yet, accurate diagnosis for low viral loads is currently lacking. Combining isothermal amplification with the CRISPR/Cas12a system not only overcomes the issue of insufficient sensitivity, but also compensates for the false-positive tendency of RPA, thus representing an effective strategy for on-site rapid detection [43]. The RPA-CRISPR/Cas12a arrays have been used for pathogen detections in mammals, fish and crustacea, including virus (monkeypox virus, Porcine parvovirus, mandarin fish ranavirus, ISKNV, acute hepatopancreatic necrosis disease, WSSV and enterocytozoon hepatopenaei), bacteria (*P. plecoglossicida*, *H. parasuis*, *S. maltophilia* and *A. hydrophila*), and parasites (*T. gondii*, *C. sinensis*, *Anaplasma marginale* and *Babesia bigemina*) [30,31,33,44,45,46,47].

As the critical element for target recognition, the sequence and concentration of crRNA were selected to ensure optimal recognition and cleavage efficiency [44]. Three crRNAs were designed based on the *mcp* sequence of LMBRaV amplified by basic-RPA. The *mcp* gene was highly conserved among LMBRaV and served as the most reliable target for molecular detection (3–5, 8–10, 12, 16, 24, 26, 32, 42, 46). Nevertheless, to ensure long-term diagnostic accuracy, we recommend periodic sequencing of the crRNA target region to monitor any potential sequence variations that may emerge. Using dual evaluation of fluorescence signal intensity and LFD color development, crRNA-2, which exhibited the highest cleavage activity, was selected. The differences in activity among the three crRNAs indicated that the secondary structure of crRNA and its complementary efficiency with the target sequence were key factors affecting Cas12a cleavage activity [43]. There were some other key elements included in the RPA-CRISPR/Cas12a array. LbCas12a protein was the executor of trans-cleavage activity and its concentration directly affected the signal intensity and background level of the detection system [48]. The ssDNA fluorescence reporter (5′-6FAM-TTATT-BHQ1-3′) was the output carrier of the fluorescence signal. Its concentration was needed to balance signal intensity and background noise [49]. The concentrations of these key reaction parameters were optimized as follows: LbCas12a protein concentration of 150 nM, crRNA-2 concentration of 200 nM, and ssDNA reporter concentration of 200 nM. Notably, when the LbCas12a concentration exceeded 200 nM, the fluorescence signal decreased, suggesting that excessively high protein concentrations might lead to non-specific adsorption or an imbalance in the reaction system [34,49,50,51]. It has been reported that in integrated nucleic acid amplification and CRISPR detection systems, Cas12a-mediated cis-cleavage competed with polymerase-mediated amplification, and excessive enzyme activity could suppress amplification efficiency, thereby reducing overall signal output [50]. Therefore, maintaining an optimal stoichiometric ratio among Cas12a, crRNA, and other reaction components was critical for achieving maximal trans-cleavage activity and signal-to-noise ratio. The reaction conditions established in this study provided a foundation for stable detection performance.

Specificity evaluation showed that the dual-recognition mechanism of RPA-CRISPR/Cas12a conferred high specificity [36]. Both detection systems specifically recognized LMBRaV and showed no cross-reactivity with other aquatic virus (CyHV-2, GSIV, ISKNV, GCRV II, CrERV, WSSV) or the largemouth bass host genome, effectively avoiding false-positive results that could arise from non-specific RPA. Sensitivity assessment revealed that both the RPA-CRISPR/Cas12a assay and the RPA-CRISPR/Cas12a-LFD visual assay achieved a detection limit of 1 copy/μL for LMBRaV, benefiting from the integrated “amplification-recognition-signal amplification” design. This performance was superior to the previously reported RPA-CRISPR/Cas12a method for LMBRaV (sensitivity of 50 copies per reaction) [39], which might be attributed to the optimized CrRNA having high efficient Cas12a cleavage activity. Additionally, the detection limit of the LMBRaV RPA-CRISPR/Cas12a assays was lower than that of previous LMBRaV detection methods, such as PCR (10^4^ copies/μL), qPCR (7.6 copies/μL), LAMP (85.5 copies/μL), TaqMan PCR (100 copies/μL), and ddPCR (2 copies/μL) [12,22,24,26,42]. A comprehensive comparison of our method with previously reported LMBRaV detection methods is summarized in Table 6. The single-copy or near-single-copy levels’ detection limits were also demonstrated for other pathogens using RPA-CRISPR/Cas12a assays [37], while some RPA-CRISPR/Cas12a assays exhibited higher detection limits. The detection limit of AHPND RPA-CRISPR/Cas12a assay was 200 copies/μL [45], monkeypox virus RPA-CRISPR/Cas12a assay was 100 copies/μL [44], ASFV RPA-CRISPR/Cas12a assay was 3 × 10^4^ copies/μL [52], and PPV RPA-CRISPR/Cas12a assay was 100 copies/μL [53]. However, direct comparisons of these values with our results should be interpreted with caution, as differences in reaction conditions, template quality, and detection formats may affect assay performance. Although the RPA-CRISPR/Cas12a assays achieved the same detection limit as previously developed RPA assays for LMBRaV [3], its characteristic of having a lower rate of false positives made it more suitable for accurate detection. Clinical sample validation revealed that RPA-CRISPR/Cas12a assay, RPA-CRISPR/Cas12a-LFD assay, and ddPCR assay achieved a 100% detection rate, which was higher than conventional PCR assay. Although the number of clinical samples in this study was relatively small and further validation with larger cohorts is needed, our findings are consistent with previous studies showing that RPA-CRISPR/Cas12a methods increased the detection rate of clinical samples by more than four times compared with PCR [42]. These data indicated that RPA-CRISPR/Cas12a assay was more suitable for monitoring low-copy viruses, such as early infection animals or asymptomatic carriers.

In addition to the high sensitivity, the test time of LMBRaV RPA-CRISPR/Cas12a assays completed the entire reaction within 45–60 min, while PCR, qPCR and ddPCR assays needed more than 1 h (Table 6). LAMP, TaqMan PCR and qPCR assays required calibration curve construction, which made them more complex. Compared to the qPCR and ddPCR, the RPA-CRISPR/Cas12a assays required no specialized instrumentation and results were read by the naked eye. Although these two RPA-CRISPR/Cas12a assays offered some advantages, a quantitative interpretation standard correlating dipstick color intensity with viral load still needed to be established. In addition, the two-step procedure carries a potential risk of aerosol contamination. However, this risk could be minimized through simple operational measures, including physical separation of pre- and post-amplification steps, dedicated pipettes with filter tips, multiple negative controls, and clean bench procedures. To facilitate practical implementation, we evaluated several key aspects of our assay. The estimated cost was approximately ¥50 per reaction. Equipment requirements were minimal: a portable metal bath, a microcentrifuge, and pipettes. The LFD format required no fluorescence detector, making it suitable for resource-limited settings. Operator training could be completed within 2–3 h, and with sufficient reaction consumables and equipment capacity, a larger number of samples could be processed simultaneously. Looking forward, the development of one-pot or closed-tube CRISPR systems represents a promising approach to further simplify the workflow and reduce contamination risk [54,55]. To address the limitation of aerosol contamination, Xie et al. developed paraffin-embedded lyophilized CRISPR/Cas12a microspheres that spatially separate the amplification and detection components [55]. The heat-triggered one-pot CRISPR systems represent a promising strategy for future improvement. Furthermore, the dependence of Cas12a on PAM sequences limited the choice of target sites. Future exploration of PAM-independent strategies (such as asymmetric RPA or kinetic/thermodynamic-based methods) might help broaden the applicability of the detection platform [56,57].

## 5. Conclusions

Based on the specific recognition and trans-cleavage mechanism of the CRISPR/Cas12a system, this study successfully established the RPA-CRISPR/Cas12a assay and the RPA-CRISPR/Cas12a-LFD visual assay for LMBRaV detection. Both methods offered high sensitivity, specificity, and simple operation for isothermal diagnostics. Clinical validation results were completely consistent with ddPCR, and the detection rates were significantly superior to those of conventional PCR. These detection systems showed potential application prospects for clinical diagnosis of LMBRaV, low virus load surveillance, on-site rapid detection in aquaculture settings, and they also provided a reference platform for the development of point-of-care testing technologies for other aquatic animal pathogens.

## Figures and Tables

**Table 1:** Sequences of crRNAs, primers, and ssDNA reporters.

Primer Name	Sequence
crRNA-1	UAAUUUCUACUAAGUGUAGAUCAACUACACUUGCGUCACUCC
crRNA-2	UAAUUUCUACUAAGUGUAGAUGAGCGGGUAACACCGUCCUGGA
crRNA-3	UAAUUUCUACUAAGUGUAGAUCGUCACUCCUGUUGUUGGAGCGG
F6	CATGTTTATGGTGCAAAACGTCACT
R6	ATAGTACTCTACGCTCATGTCTGGAAG
LFD ssDNA reporter	6FAM-TTATT-Biotin
ssDNA Reporter	6FAM-TTATT-BHQ1

Note: All three selected crRNAs showed no significant off-target hits and had favorable secondary structure predictions (ΔG values: −1.2 to −2.8 kcal/mol).

**Table 2:** RPA-CRISPR/Cas12a detection system.

Component	Volume (μL)	Final Concentration
10× HOLMES Buffer for Cas12a	2	1×
10 μM LbCas12a Nuclease	0.5	25–250 nM
10 μM crRNA	0.5	25–250 nM
Basic-RPA product (target DNA)	1	—
10 μM ssDNA reporter	0.5	25–250 nM
Nuclease-free water	15.5	—
Total volume	20	—

**Table 3:** RPA-CRISPR/Cas12a-LFD detection system.

Component	Volume (μL)	Final Concentration
10× HOLMES Buffer for Cas12a	2	1×
10 μM LbCas12a Nuclease	0.5	25–250 nM
10 μM crRNA	0.5	25–250 nM
Basic-RPA product (target DNA)	1	—
10 μM LFD-ssDNA reporter	0.5	25–250 nM
Nuclease-free water	15.5	—
Total volume	20	—

**Table 4:** Clinical samples validation.

Number	PCR	RPA-CRISPR/ Cas12a	RPA-CRISPR/ Cas12a-LFD	Viral Copy Number by ddPCR (Copies/μL)
1	+	+	+	12,670
2	+	+	+	7820
3	-	-	-	0
4	+	+	+	19,000
5	+	+	+	4950
6	+	+	+	1710
7	+	+	+	1740
8	+	+	+	760
9	+	+	+	27,500
10	-	-	-	0
11	+	+	+	1060
12	-	-	-	0
13	+	+	+	1730
14	+	+	+	1000
15	-	-	-	0
16	-	+	+	56
17	-	-	-	0
18	-	+	+	14
19	-	+	+	8
20	-	-	-	0
21	+	+	+	94,000
22	-	-	-	0
23	-	-	-	0
24	+	+	+	92,000
negative control	-	-	-	0

+: positive; -: negtive.

**Table 5:** Detection of LMBRaV in clinical samples using four methods.

Comparison of the Methods	Total	Positive Samples	Negative Samples	Positive Rate	Detection Rate
PCR	24	13	11	54.2%	81.26%
RPA-CRISPR/Cas12a	24	16	8	66.7%	100%
RPA-CRISPR/Cas12a-LFD	24	16	8	66.7%	100%
ddPCR	24	16	8	66.7%	100%

**Table 6:** Comprehensive comparison of the developed method with previously reported LMBRaV detection methods.

Method	Target Gene	Detection Limit (Copy/μL)	Assay Time	Equipment Requirement	Workflow Steps	Reference
RPA-CRISPR/Cas12a	*mcp*	1	45–60 min	metal bath	Two-step	This study
RPA-CRISPR/Cas12a-LFD	*mcp*	1	45–60 min	metal bath + qPCR instrument	Two-step	This study
RPA-CRISPR/Cas12a	*mcp*	50	60 min	metal bath + qPCR instrument	Two-step	[39]
RPA-LFD	*mcp*	89	<30 min	metal bath	One-step	[26]
ddPCR	*mcp*	2	3 h	ddPCR instrument	Multi-step	[24]
qPCR	*mcp*	7.6	2 h	qPCR instrument	One-step	[22]
TaqMan qPCR	*mcp*	100	2 h	qPCR instrument	One-step	[12]
LAMP	*mcp*	85.5	1 h	water bath	One-step	[21]
PCR	*mcp*	10^4^	2.5 h	PCR instrument	Multi-step	[42]

## Data Availability

The original contributions presented in this study are included in the article. Further inquiries can be directed to the corresponding author.

## Supplementary Materials

The following supporting information can be downloaded at: https://www.mdpi.com/article/10.3390/vetsci13080825/s1. Figure S1. Schematic diagram for crRNA design; Figure S2. Detection results of clinical samples using the RPA-CRISPR/Cas12a-LFD assay (a), the RPA-CRISPR/Cas12a assay (b), and conventional PCR (c). M: DL1000 marker, NC: nuclease-free water.
